# Exploring the Feasibility of Using ChatGPT to Create Just-in-Time Adaptive Physical Activity mHealth Intervention Content: Case Study

**DOI:** 10.2196/51426

**Published:** 2024-02-29

**Authors:** Amanda Willms, Sam Liu

**Affiliations:** 1 School of Exercise Science, Physical and Health Education University of Victoria Victoria, BC Canada

**Keywords:** ChatGPT, digital health, mobile health, mHealth, physical activity, application, mobile app, mobile apps, content creation, behavior change, app design

## Abstract

**Background:**

Achieving physical activity (PA) guidelines’ recommendation of 150 minutes of moderate-to-vigorous PA per week has been shown to reduce the risk of many chronic conditions. Despite the overwhelming evidence in this field, PA levels remain low globally. By creating engaging mobile health (mHealth) interventions through strategies such as just-in-time adaptive interventions (JITAIs) that are tailored to an individual’s dynamic state, there is potential to increase PA levels. However, generating personalized content can take a long time due to various versions of content required for the personalization algorithms. ChatGPT presents an incredible opportunity to rapidly produce tailored content; however, there is a lack of studies exploring its feasibility.

**Objective:**

This study aimed to (1) explore the feasibility of using ChatGPT to create content for a PA JITAI mobile app and (2) describe lessons learned and future recommendations for using ChatGPT in the development of mHealth JITAI content.

**Methods:**

During phase 1, we used Pathverse, a no-code app builder, and ChatGPT to develop a JITAI app to help parents support their child’s PA levels. The intervention was developed based on the Multi-Process Action Control (M-PAC) framework, and the necessary behavior change techniques targeting the M-PAC constructs were implemented in the app design to help parents support their child’s PA. The acceptability of using ChatGPT for this purpose was discussed to determine its feasibility. In phase 2, we summarized the lessons we learned during the JITAI content development process using ChatGPT and generated recommendations to inform future similar use cases.

**Results:**

In phase 1, by using specific prompts, we efficiently generated content for 13 lessons relating to increasing parental support for their child’s PA following the M-PAC framework. It was determined that using ChatGPT for this case study to develop PA content for a JITAI was acceptable. In phase 2, we summarized our recommendations into the following six steps when using ChatGPT to create content for mHealth behavior interventions: (1) determine target behavior, (2) ground the intervention in behavior change theory, (3) design the intervention structure, (4) input intervention structure and behavior change constructs into ChatGPT, (5) revise the ChatGPT response, and (6) customize the response to be used in the intervention.

**Conclusions:**

ChatGPT offers a remarkable opportunity for rapid content creation in the context of an mHealth JITAI. Although our case study demonstrated that ChatGPT was acceptable, it is essential to approach its use, along with other language models, with caution. Before delivering content to population groups, expert review is crucial to ensure accuracy and relevancy. Future research and application of these guidelines are imperative as we deepen our understanding of ChatGPT and its interactions with human input.

## Introduction

Physical inactivity is a key modifiable risk factor for many chronic conditions, including cardiovascular disease, type 2 diabetes, and cancers, throughout the lifespan [1]. Despite this evidence, adults and adolescents alike are not consistently meeting the recommended guidelines to prevent developing these chronic conditions [2]. Previous studies have shown that 150 minutes of moderate-to-vigorous physical activity (MVPA) can reduce the risk of all-cause mortality by at least 30%, along with reducing the risk for chronic conditions such as cardiovascular disease (30%), colon cancer (20%), and breast cancer (14%) [3]. Although many chronic diseases affect adults, healthy lifestyle habits need to be developed early from childhood. Children aged 8 to 12 years are more flexible than adults in their ability to change behaviors because they are just beginning to develop self-regulation skills, habits, and identities for healthy living [4,5]. Thus, many countries such as Canada [6], the United States [7], and the United Kingdom [8] have set guidelines recommending 60 minutes of MVPA per day for children 17 years and younger [2]. However, despite these recommendations, physical inactivity is prevalent among children, with less than one-quarter of children meeting the guidelines in countries such as Canada [9] and the United States [9]. Consequently, promoting regular PA to prevent chronic diseases and maintain lifelong health has been a key priority for governments worldwide.

Recent studies suggest that family-based PA programs can be an effective strategy to improve PA levels in children [10,11]. These programs focus on providing guidance for parents to support their child’s PA (eg, encouragement, providing opportunity, and logistic support) [12]. With advancements in mobile health (mHealth) technologies and improved access to smartphones, emerging evidence indicates that PA interventions delivered through mHealth technology can be effective while improving scalability and personalization. However, the effectiveness and engagement of interventions vary depending on the intervention design and the degree of tailoring [13,14]. Studies have demonstrated that tailored mHealth interventions are more effective in improving behavior and health outcomes compared with nontailored interventions [15]. A recent advancement in tailored mHealth interventions is the development of just-in-time adaptive interventions (JITAIs), which use mHealth technology to assess the dynamically changing needs of individuals and deliver tailored support in real time [14,16]. Thus far, JITAIs have shown great promise in promoting PA among adults [17], university students [18], and chronic disease populations [19]. Further, innovative mHealth “no-code” development platforms, such as Pathverse, have made the development and implementation of JITAIs much easier and cost-effective [20,21]. However, the development of content for JITAIs can be extremely labor-intensive due to the need to create various versions of health-related content for different tailored algorithms. Although the documentation of content creation timelines for PA JITAIs is in its infancy, a typical timeline for PA content creation from the formative phase to pilot testing reportedly ranges from 12 [22] to 15 months [23,24].

Specific to JITAIs, the typical process of creating evidence-based and engaging content for these mHealth interventions typically involves the following steps [21,25]: (1) defining the behavior change theories and behavior change techniques (BCTs) required for the intervention [26]; (2) gathering evidence from various sources, such as previous literature, public health sources, gray literature, and blogs, and then adapting it to suit the needs of the intervention and deliver it through the chosen medium; and (3) writing content that is engaging and matches the literacy level of the target population for the app. These steps can often be time-consuming, with the need for researchers to follow these steps iteratively and repetitively for the duration of the design of the intervention. Further, despite the consideration of these steps, several challenges still arise in the development of JITAI content. Existing studies have identified limitations, such as the need for more extensive content within interventions, struggles in creating novel and meaningful messages, and challenges in tailoring messages to diverse user preferences [27,28]. These studies have also recognized the resource constraints in developing content to meet these needs and the complex, multidimensional nature of creating tailored and engaging content for their sample. Therefore, an artificial intelligence (AI) tool such as ChatGPT (OpenAI) [29] can be extremely useful in making the process of generating JITAI content for mHealth interventions faster and more cost-effective. ChatGPT offers a solution to the need for more content within interventions by leveraging its vast training data and the ability to generate a diverse set of messages efficiently. Further, its generative capabilities and the ability for users to continually prompt new rules address the challenge of creating novel content, reducing the risk of messages being perceived as overly simplistic.

ChatGPT was first launched by OpenAI in November 2022 and is an open AI language model that generates human-like responses to text-based prompts [30]. It can understand and generate responses in various languages, as well as debug code, write stories in different genres and lengths, summarize information from complex texts, offer explanations on various topics, and even reject answering inappropriate prompts [31]. Unlike other generative large language models (LLMs), ChatGPT stands out as the inaugural member of a series of highly scaled LLMs that attain state-of-the-art performance with minimal need for fine-tuning [32]. Further, ChatGPT is highly sophisticated in that it is able to provide continuous dialog by remembering what the user has said earlier in the conversation thread [33].

Although ChatGPT hosts an impressive suite of features and capabilities, there are also several ethical and privacy concerns to keep in mind while using this service. First, it is important to note that ChatGPT “learns” its information from human input. This is subject to error and is limited based on what others have input into its system. Further, when generating health information content, in particular, this LLM has been extensively trained with data up to 2021, thus limiting some of the relevance and accuracy of current practices [34]. Second, ChatGPT stores its data in the United States, which, depending on the type of information being input into the United States, may be subjected to privacy concerns based on US freedom and privacy laws. To build on this consideration of data storage, it is crucial not to input any personal health information or other sensitive data into ChatGPT, as this LLM continues to learn from text prompts.

Since its inception, ChatGPT has been widely cited in various bodies of behavioral science literature as a virtual assistant, chatbot, and language translation tool [35]. To generate output from the program, a concept called prompt engineering is one method that explains how ChatGPT generates output [36]. In LLMs, a prompt is defined as an instruction to the model that customizes, enhances, or refines the output [37]. However, there is currently a lack of studies examining the feasibility of using ChatGPT to help develop intervention content for JITAIs aimed to promote PA when given a behavior change theory and a behavior target outcome.

Thus, the primary objective of this paper was to present an autoethnographic case study that explored the feasibility, including the acceptability and ease of use, of using ChatGPT to create content for a family-based PA JITAI mobile app. The secondary objective was to describe lessons learned and future recommendations for using ChatGPT in developing mHealth intervention content.

## Methods

### Study Design

This case study consisted of 2 phases, which took place from March 1, 2023, to April 30, 2023. In phase 1 (0-2 months), we used ChatGPT-3 to develop a 10-week family-based PA JITAI. In phase 2 (3-4 months), we described lessons learned based on our experience of using ChatGPT in phase 1 and provided future recommendations for using ChatGPT in the development of mHealth interventions.

### Ethical Considerations

This paper outlines the procedural aspects of using ChatGPT for content generation for a subsequent study. Given that it operates independently without involvement of human participants or sensitive data, formal ethics approval from our institution was deemed unnecessary.

### Phase 1

We explored the feasibility of using ChatGPT to create content for the PA JITAI mobile app. To determine the feasibility of using ChatGPT to rapidly create JITAI content, we used an autoethnographic case study approach [38]. This method enabled the researchers (AW and SL) to reflect on their experience of using ChatGPT. While using ChatGPT, the researchers created field notes and had a meeting to discuss their independent experiences with using ChatGPT-3. Specifically, we reflected on the acceptability and ease of use as key areas of focus for feasibility [39]. Results of the meeting were themed into acceptability and ease of use of using ChatGPT. Assessing acceptability metrics involved reflecting on the satisfaction of the response generated by ChatGPT. The ease-of-use assessment involved reflecting on ChatGPT usability [39]. In this phase, we used 2 tools, Pathverse and ChatGPT. Pathverse is a no-code app builder platform that supports mHealth research [20,40]. It consists of a web portal for researchers to create engaging mobile app interventions with “drag and drop” features instead of coding. The content is then instantly displayed on the Pathverse mobile app. We used ChatGPT-3 to generate the content needed to be added to Pathverse. To gather feasibility data, we generated intervention content to support parents to help their child (8-12 years of age) to be physically active.

**Figure 1:**
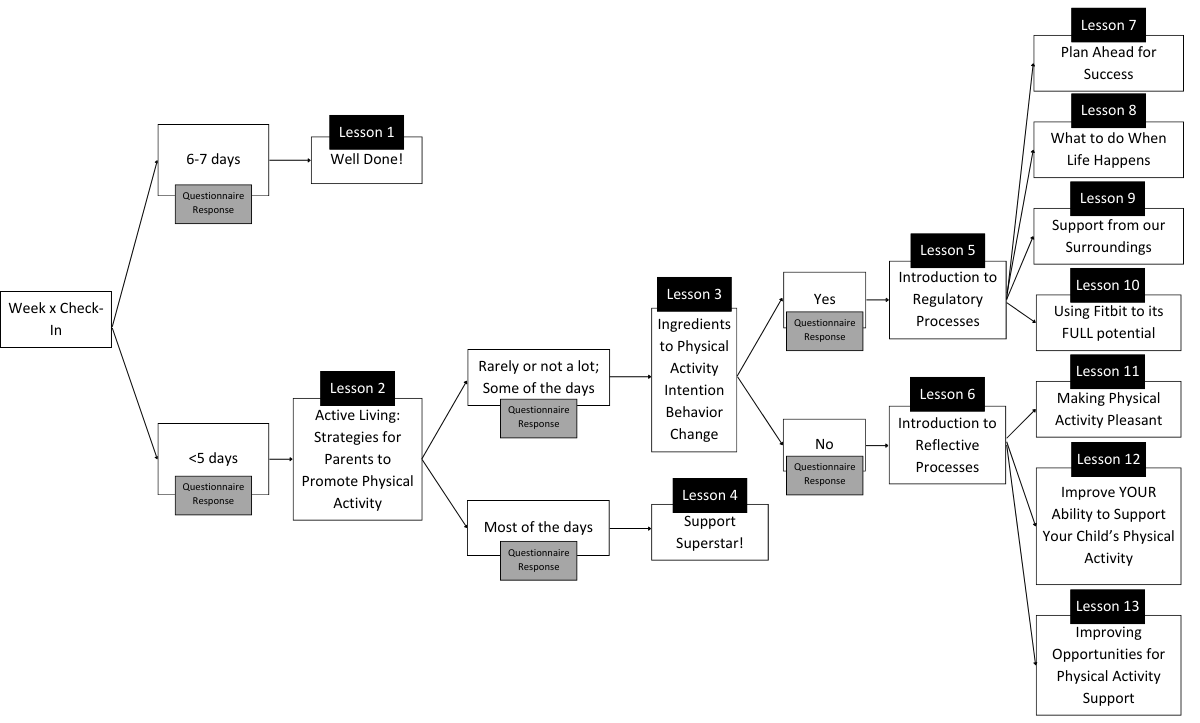
Names of modules in the decision tree algorithm for personalized lessons.

The content generated for this app was developed based on the Multi-Process Action Control (M-PAC) framework [41,42]. The M-PAC framework addresses the intention-behavior gap through the understanding that ongoing reflective processes (ie, affective attitude and perceived opportunity) and regulation processes (ie, behavioral and cognitive tactics to maintain intention focus) are necessary for the intention to become an action [41]. Specific to a JITAI, the M-PAC framework was selected as the framework for this intervention to dynamically and contextually address users’ failed intentions to be physically active. Thus, the just-in-time intervention options can be tailored to the specific circumstances of the individual, aligning with either the reflective, regulatory, or reflexive process [41,42]. The M-PAC framework was additionally chosen as we have seen success with this framework and its associated BCTs (ie, action planning, repetition, and habit formation) in previous family-based PA programs [43]. To address these circumstances, our research team created decision tree algorithms to tailor the family lessons and challenges recommended throughout the weeks. The algorithms were designed using the M-PAC framework and take into consideration (1) child MVPA minutes, (2) parent support behavior, and (3) parent self-efficacy and motivation for supporting their child’s PA (Figure 1). Based on the decision tree, weekly tailored lessons needed to be created to target each M-PAC construct. Topics included parental support, affective attitudes toward supporting their children’s PA, capability, opportunity, self-monitoring of PA, and restructuring the environment for PA. These topics stemmed from previous research for family-based PA interventions using the M-PAC framework [43]. With these considerations, a variety of prompts were created based on these topics.

There are various components to consider when generating a prompt for ChatGPT. Specific to academic uses of ChatGPT, the elements to be included in a prompt include an instruction (ie, an overview of the output you would like to receive), context (ie, other background information to help tailor the output), input data (ie, additional specifications for the output that may include its strengths or limitations), and output indicator (ie, how you would like the output to be presented, including word count and paragraph format) [44]. When creating a prompt for this case study, we included the target behavior and the M-PAC framework, with each output to be delivered in bullet point form. Once the content was created, we then used the Pathverse mHealth no-code app design tool to develop the JITAI app [20,38-40]

### Phase 2

We summarized lessons learned and future recommendations for using ChatGPT in the development of mHealth interventions. Our team identified common themes and patterns emerging from the process of creating the JITAI content using ChatGPT. We then compared our data with previous literature to develop recommendations for future use. This involved a literature search to identify relevant studies and lessons learned from using ChatGPT in mHealth interventions. The primary aim of the literature search was to gather a wide range of insights into the acceptability, including the application of ChatGPT and its effectiveness in this context and challenges associated with integrating ChatGPT into mHealth interventions to refine our recommendations.

## Results

### Phase 1: Exploring the Feasibility of Using ChatGPT to Create JITAI Content

**Figure 2:**
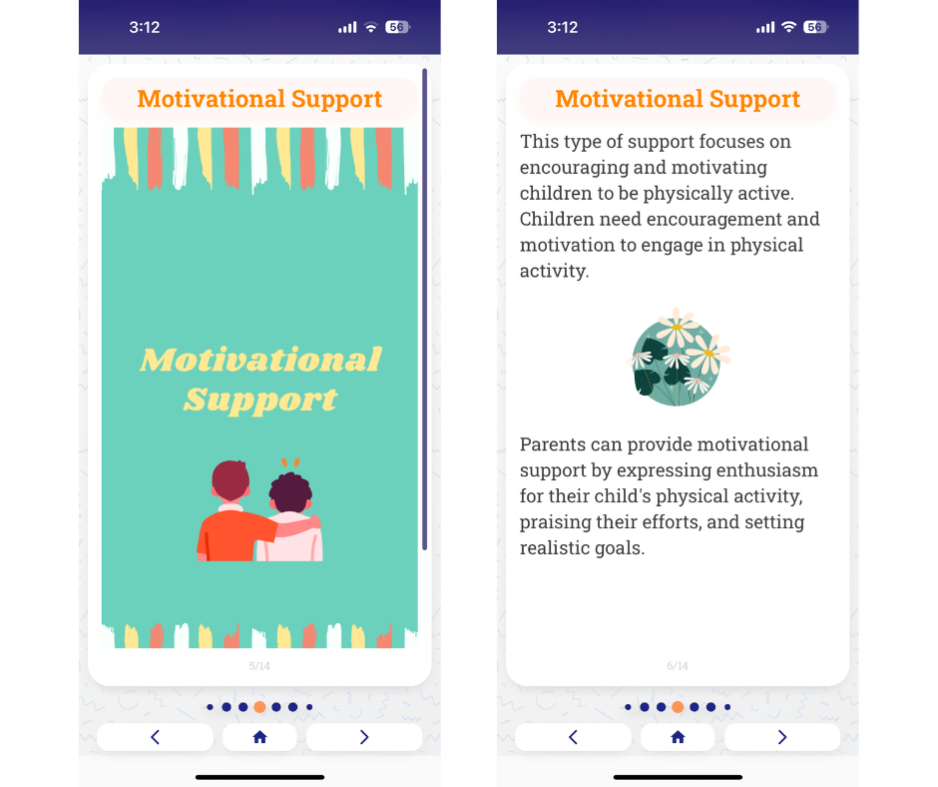
Screenshots of physical activity content generated by ChatGPT in the mobile app Pathverse.

The results of phase 1 are first reported on how the researchers (AW and SL) used ChatGPT to generate content, followed by an analysis of the feasibility of the use of ChatGPT in this context. Overall, we created 13 lessons with the help of ChatGPT in phase 1. Figure 2 displays an example of how this content was displayed in the mobile app. We provided specific prompts about the length of the content generated, the target constructs of the M-PAC framework, the tone of the lesson, and the literacy levels needed. We used multiple question prompts to optimize text output. Table 1 provides examples of prompts used for different lessons. We started with broad prompts (eg, explain the various constructs in the M-PAC framework) and then used specific prompts based on the output (eg, provide specific fun examples to help parents improve opportunities to support child PA; Table 1). After the prompts were input into ChatGPT, the output was copied into a separate document for review by the researchers (AW and SL). If more or alternate content was needed, prompts such as “provide additional information about [this topic]” were used. To ensure that the output given by ChatGPT was relevant and accurate, we referred to previous literature and previous content examples following the M-PAC framework [21,45,46]. Once the content was deemed acceptable and accurate by the researchers, it was uploaded to the Pathverse platform. This step additionally involved creating graphics to include along with the text responses and formatting the content into different app “pages” with fewer than 400 characters per page of the mobile app.

We evaluated the acceptability of ChatGPT for creating mHealth content by reflecting on content accuracy, relevance, and tone. Both researchers found that ChatGPT demonstrated an acceptable level of accuracy and relevance and provided relevant responses to the prompts. However, on some occasions, ChatGPT provided false academic references. This is a serious issue that needs to be addressed to prevent misinformation. Thus, both authors reflected the need to place a filtering mechanism to ensure that the content generated was appropriate. Furthermore, some of the answers lacked specificity (eg, provide examples of PA programs in my area). This may be due to the fact that ChatGPT-3 was trained using data up to September 2021. Finally, we found the tone of ChatGPT responses to be acceptable for research purposes. The overall tone matched the prompt given (eg, write in a fun and positive voice). Overall, ChatGPT did not generate any inappropriate content. There is an evident need to provide clear prompts in order for ChatGPT to provide optimal responses. Additionally, multiple questions are often needed to optimize ChatGPT responses. The researchers additionally agreed that providing a role to ChatGPT, for example, telling the LLM that it is a health researcher delivering a family-based PA intervention, may have further refined the tone and quality of the response given.

When reflecting on the feasibility of implementing ChatGPT for this case study, we (AW and SL) found ChatGPT to be easy to use. Both researchers (AW and SL), with varying levels of technical expertise, found the user interface to be intuitive. The ease of use also allowed us to test various prompts to help optimize the ChatGPT responses. Overall, we found that minimal training or prior experience is needed to use this tool, and it has the potential to make it widely accessible for researchers.

**Table 1:** Prompts and responses from a conversation with ChatGPT in generating content for a 10-week family-based physical activity mobile app.

Prompt entered	ChatGPT response
Write a fun to read article about the role of parental capabilities to support for child physical activity based on the M-PAC^a^ framework	Are you struggling to find ways to support your child’s physical activity? Do you want to improve your parental capabilities to make physical activity a fun and regular part of your child’s life? Look no further than the M-PAC framework, which provides a roadmap for parents to support their child’s physical activity. The M-PAC framework stands for Multi-Process Action Control, and it emphasizes the importance of three key components for parents to successfully support their child’s physical activity: instrumental support, regulatory support, and motivational support. Instrumental support refers to the tangible resources and skills that parents provide to support their child’s physical activity. This could include things like providing transportation to and from activities, purchasing sports equipment, or enrolling your child in a physical activity program. Regulatory support focuses on creating an environment that supports physical activity. This could include setting rules and boundaries around screen time, creating a routine for physical activity, or providing healthy food options to fuel physical activity. Motivational support is all about fostering a positive attitude towards physical activity. This could include providing praise and encouragement, setting goals, and finding ways to make physical activity fun and enjoyable.
Provide specific fun examples to help parents improve their capabilities for supporting child physical activity in bullet points	Certainly! Here are some specific fun examples to help parents improve their capabilities for supporting child physical activity: Instrumental Support: Have a weekly family game night that involves physical activity such as charades, Pictionary or dance party. Organize a scavenger hunt with physical activity challenges for your child and their friends. Plan a family hike or outdoor adventure at a nearby park or nature reserve. Regulatory Support: Start a vegetable garden together and teach your child the importance of physical activity and nutrition for a healthy lifestyle. Use a fitness tracker or pedometer with your child to encourage them to walk more and set goals for daily steps. Plan a family bike ride or roller skating adventure on a weekend afternoon. Motivational Support: Make a fun reward system for reaching physical activity goals, such as a family outing to a local amusement park or water park. Watch sports games or videos of active events with your child and talk about the benefits of physical activity for overall health. Encourage your child to invite friends over for a game of soccer, basketball or other team sport to promote socialization and physical activity. By incorporating these fun examples into your family’s routine, you can improve your capabilities for supporting your child’s physical activity and make healthy habits a fun and rewarding part of your family’s lifestyle.

^a^M-PAC: Multi-Process Action Control.

### Phase 2: Recommendations for the Future Use of ChatGPT

#### Overview

On the basis of our experience in developing the above content and based on previous research [21,25,47], we have compiled the following recommendations for using ChatGPT for similar purposes. First, a 6-step recommendation was proposed to help make the process of using ChatGPT more efficient for future research. These steps included defining the target behavior for the intervention, applying behavior change theory, creating the intervention structure, inputting this information into ChatGPT, refining the output, and customizing the content to be delivered to the target population. Second, we discussed ethical considerations for the use of ChatGPT in this research space. Lastly, we discussed transparency with using ChatGPT in academic research.

#### Step 1: Determine Target Behavior

The first step of these guidelines involves determining a target behavior or behaviors for the target population of your intervention. This could be based on previous research for certain demographic groups or based on a gap in the current literature. The selected target behavior defines the purpose of the intervention and the outcomes on which the intervention will be assessed [25]. This is considered an essential first step as it will guide the remaining steps of these recommendations.

#### Step 2: Ground in Behavior Change Theory

The second step recognizes the need to deliver digital health content grounded in behavior change theory. Based on previous literature and considering the target behavior selected in step 1, it is advised to select a health behavior theory to guide the intervention. Thus, constructs of the behavior change theory must be considered when searching for and developing digital health intervention content. Further, other elements of the intervention, such as BCTs, to strengthen the behavior change theory [26] should be considered during this step.

#### Step 3: Design Intervention Structure

Step 3 involves designing the intervention structure. In this step, the length of the intervention and the length and amount of content to be delivered should be considered first. After this information has been determined, it is recommended to consider the medium of delivery of the digital health intervention content. Previous research has shown varying success for both web-based interventions and mobile-based interventions [48,49]. Additionally, there are important considerations for best practices with delivering content through these different mediums, which are explored later in this development process.

It is important to note that this step may involve an additional agenda. Examining previous literature, using participatory action research or co-design principles, or other methods may be necessary to ensure that you are gathering content that will be both engaging to the participants and promoting adherence to the target behavior.

#### Step 4: Input Intervention Structure and Behavior Change Constructs Into ChatGPT

The next step is to input the information gathered from steps 1 to 3 and create varying prompts into ChatGPT. If this is your first time logging into ChatGPT through OpenAI, you will need to create a free account. Once your account has been created, you may type your prompt into the text box at the bottom of the screen. Determining an optimal prompt to input includes considering the target behavior, the proposed structure of the intervention, the behavior change theory and its constructs, and BCTs. Further, it is important to consider the rules in which ChatGPT delivers its output, for instance, whether you would prefer the response to be in paragraph form or bullet points. This step is iterative as you receive responses and continue to modify your prompt until you receive the desired output. Additionally, it has been previously recommended to consider assigning a role and tone for ChatGPT to embody in its response or to provide a similar example, when available [50].

#### Step 5: Revise the Output of ChatGPT

This step involves revising the response received from ChatGPT. There is a possibility that the language model has created errors or has provided incorrect references with their output. We compared the results with previous literature and revise and adapted as necessary to ensure that the most accurate information is being provided. Including information from the previous literature in the next prompt may continue to provide more refined ChatGPT responses.

#### Step 6: Customize the Content to be Delivered

The final step of this framework is to customize the content to meet the needs of your intervention. This involves considering the layout and design of how you will deliver the content on your selected medium from step 3, as well as any images or graphics used to supplement the given content. This step may involve working with an additional team to develop a web-based or mobile-based platform to support the health behavior change intervention. Further, user experience and design should be considered to improve usability and satisfaction of the content [51-54]. Table 2 summarizes the steps of these guidelines and considerations to meet the needs of each step.

By following these guidelines and using ChatGPT to assist in the rapid creation of digital health content, many ethical considerations arise. The first consideration, as highlighted above, is ensuring that the responses from ChatGPT are accurate and validated to be used as health information in a research study. This can be done by referencing previous literature or creating a panel of experts in the field to review the output created by ChatGPT. Further, it is vital to ensure that users engaging with AI-generated content through ChatGPT or other LLMs are adequately informed about its limitations, decision-making capabilities, and the crucial nature of their involvement. Transparent communication and obtaining informed consent are pivotal to respect user autonomy and comprehension. Although ChatGPT demonstrates remarkable efficiency in generating responses to prompts, evaluating its applicability within the intervention’s context remains crucial to ensure substantial value to using ChatGPT.

As ChatGPT inevitably continues to support academic research across disciplines, it is also important to consider how ChatGPT is being cited by those who use it. There has been a variety of techniques used so far, with some authors including ChatGPT as an author [55] and others acknowledging the use of ChatGPT [34] to assist with their manuscript.

**Table 2:** Proposed recommendations for developing digital health content using ChatGPT and a summary of considerations for using this tool.

Step	Task	Consideration
1	Determine target behavior	Previous research Needs of the target population
2	Ground in behavior change theory	Stage of readiness of participants Needs of the population group
3	Design the intervention structure	Web or mobile based Length of the intervention Amount of content to be delivered in each bout (ie, how many words, characters, or pages of content to be delivered) Use co-design or other frameworks to ensure that the intervention aligns with the needs of the target population
4	Input intervention structure and behavior change constructs into ChatGPT	Structure prompt to input into ChatGPT (considering instruction, context, input data, and output indicator) Iteratively adapt prompts based on desired output Order in which relevant information relating to each construct is delivered, if not predefined by the literature
5	Revise the output of ChatGPT	Refer outputs to previous literature to ensure accuracy Confirm whether references used by ChatGPT are accurate
6	Customize the content to be delivered	Layout and design of content Images or graphics to supplement text output User experience and design of the intervention platform

## Discussion

### Principal Findings

The primary objective of this study was to explore the feasibility of using ChatGPT to develop content for a mobile-based JITAI to promote parental support for their children’s PA. The secondary objective was to propose recommendations for using ChatGPT for future work in this area. To our knowledge, the process of using ChatGPT to develop health intervention content has not yet been documented, so we considered the key components required to develop effective behavior change interventions. We found that using ChatGPT was overall acceptable for this case study. However, a human check by researchers in the field is imperative to ensure the relevance and accuracy of the output provided. The use of ChatGPT and similar LLMs is rapidly evolving, and as such, these proposed recommendations are highly dynamic to the developing nature of these technologies.

This study has several implications for researchers using ChatGPT when developing mHealth app content. First, ChatGPT can help researchers improve the efficiency of creating digital health content for various tailored lessons. Previously, it was determined that ChatGPT can expedite the research process by allowing researchers to focus on steps of the research design process that require more human input, for example, focusing on the experimental design [56,57]. The improvement and versatility of text generation, knowledge translation, and literature review have been documented in various studies that have used ChatGPT in health care education [58]. As seen in this study, ChatGPT can help create various versions of content (varying in writing styles and tones) using a series of different prompts. Further, coupled with the efficiency of developing intervention content, this study has highlighted the ability to efficiently create a variety of tailored content specific to PA messaging. The need for more variety and content options has been previously stated as a limitation in previous studies that did not use ChatGPT for the creation of content [28]. Overall, this study highlights one use case that benefited from the use of ChatGPT to rapidly create digital health content. As ChatGPT is in its infancy, we expect it to evolve quickly [58].

Second, this study highlights the current limitations of using ChatGPT for creating mHealth behavior interventions. Although ChatGPT has great potential to improve the efficiency with which digital health content creation can occur, it is not possible to replicate responses by ChatGPT while using the same prompt [58,59]. This unpredictability poses a significant challenge for health researchers and developers who may require stable and reliable outputs [58]. Because of the probabilistic nature of ChatGPT and similar LLMs, the responses generated from ChatGPT are generated based on a probability distribution, meaning the same response will not be generated [60]. Further, a significant concern is the generation of references by ChatGPT that do not exist or are inaccurate. This lack of interpretability hampers the transparency of mHealth content development, making it difficult for researchers to have a clear understanding of the AI’s decision-making process. Other limitations have been recognized by previous work around ChatGPT. These include limited accuracy, bias and limitations of data, lack of context, and the potential of limited engagement with the content [34]. To mitigate these challenges, we highly recommend a rigorous human fact-checking process, as indicated in our recommendations for mHealth intervention content development using ChatGPT, and fine-tuning specific prompts to ensure that the information given by ChatGPT is relevant.

Finally, the integration of ChatGPT with existing mHealth app development tools, such as Pathverse, holds the potential to significantly enhance the efficiency and effectiveness of developing and evaluating JITAI apps. By incorporating ChatGPT’s language generation capabilities into Pathverse, developers can expedite the creation of content-rich JITAIs. Additionally, reinforcement learning algorithms can play a crucial role in JITAIs by dynamically adapting the intervention based on real-time data and user feedback [61]. Developers can leverage ChatGPT’s language generation capabilities using its application programming interface to assist with content creation [61]. With the integration of ChatGPT, these algorithms can benefit from the AI-generated content to offer more tailored and contextually relevant interventions. By combining the strengths of reinforcement learning and ChatGPT, JITAI apps can become more adaptive and responsive to individual user’s needs, thereby increasing their effectiveness in promoting behavior change and improving health outcomes.

There are several limitations to this study. First, we used ChatGPT to create content for only 1 JITAI, potentially restricting the generalizability of the study findings. Second, because of ChatGPT’s tendency to provide different responses for the same prompt, it was challenging to accurately characterize the content’s reproducibility and consistency. Lastly, as ChatGPT is rapidly evolving, the use case described in this study may have limited applicability a few years from now. We also want to add that although ChatGPT-3 is currently free to use, it is likely that as it improves, it is likely to come with an associated cost.

### Conclusions

By using ChatGPT, we were able to expedite the process of creating 13 lessons that were guided by the M-PAC framework, thus highlighting the incredible opportunity ChatGPT presents to rapidly create content for various mHealth JITAIs. Although we found that ChatGPT was acceptable for this case study, we still encourage the cautious use of ChatGPT and other LLMs in similar contexts. The use of ChatGPT expedited the process of content development to 2 months, the bulk of which was spent on reviewing the content by experts in the field before delivering to population groups. This process was imperative to ensure that accurate and relevant content was being created to be delivered. The results from this study found implications in 3 areas. The first is efficiency in generating a variety of content based on different prompts. Second, this study highlighted the potential limitations of ChatGPT, including the inability to replicate responses from the same prompts and the need for human input to ensure that the output from ChatGPT is accurate. Finally, this case study has highlighted the efficiency of using no-code app builders, such as Pathverse, to disseminate information generated by ChatGPT. It is without a doubt that as ChatGPT and other LLMs continue to improve in sophistication and accuracy, they will continue to integrate into intervention design and other various contexts for researchers. Further research and applications of ChatGPT and the guidelines proposed in this study are imminent in this field as we continue to understand ChatGPT.

## Appendix

Multimedia Appendix 1 ChatGPT Transcript.

## Abbreviations

AI: artificial intelligence
BCT: behavior change technique
JITAI: just-in-time adaptive intervention
LLM: large language model
mHealth: mobile health
M-PAC: Multi-Process Action Control
MVPA: moderate-to-vigorous physical activity
PA: physical activity
