# Supplementary material for: Exploring the Feasibility of Using ChatGPT to Create Just-in-Time Adaptive Physical Activity mHealth Intervention Content: Case Study
Source: JMIR Med Educ. 2024 Feb 29;10:e51426. doi: 10.2196/51426 (PMC10940976; doi:10.2196/51426)
Supplement: Multimedia Appendix 1 [file mededu_v10i1e51426_app1.pdf]

ChatGPT Transcript

Please note that this document contains the transcripts from 6 ChatGPT threads. Not all content given from ChatGPT was used as intervention content.

<table>
<tr><td>Author</td><td>Explain the Multi-Process Action Control Framework</td></tr>
<tr><td>ChatGPT</td><td>The Multi-Process Action Control (M-PAC) framework is a theoretical model used in psychology and cognitive science to explain how individuals can regulate their behavior across a variety of situations. The framework proposes that individuals use multiple cognitive processes to control their behavior, and these processes operate at different levels of awareness and control.

The M-PAC framework consists of three key components:
1. Task representation: This component involves how individuals represent and interpret the task at hand. It includes factors such as the goals and rules associated with the task, as well as the context in which the task is performed.
2. Monitoring and control: This component involves how individuals monitor their behavior and make adjustments based on feedback and other environmental cues. It includes processes such as error detection, attentional control, and response inhibition.
3. Implementation: This component involves how individuals execute their actions based on their task representation and monitoring and control processes. It includes processes such as response selection, motor planning, and execution.

The M-PAC framework suggests that these components operate in an integrated manner to regulate behavior, and that individuals can adapt their behavior to changing situations by adjusting these components. For example, if a person is performing a task that requires sustained attention, they may use monitoring and control processes to regulate their attention and avoid distractions. If they encounter unexpected obstacles, they may adjust their task representation to incorporate new information and modify their behavior accordingly.

Overall, the M-PAC framework provides a comprehensive model for understanding how individuals regulate their behavior, and has been used to explain a wide range of cognitive and behavioral phenomena in both laboratory and real-world settings.</td></tr>
<tr><td>Author</td><td>What is the regulation process within the M-PAC framework?</td></tr>
<tr><td>ChatGPT</td><td>The M-PAC framework is a theoretical model that describes the cognitive processes involved in regulating behavior across a variety of situations. The</td></tr>
</table>

	framework includes three key components, which are task representation, monitoring and control, and implementation.<br><br>1. Task representation: This component involves how individuals represent and interpret the task at hand. It includes factors such as the goals and rules associated with the task, as well as the context in which the task is performed. The task representation component helps individuals understand what they are trying to accomplish and what strategies they can use to achieve their goals.<br>2. Monitoring and control: This component involves how individuals monitor their behavior and make adjustments based on feedback and other environmental cues. It includes processes such as error detection, attentional control, and response inhibition. The monitoring and control component helps individuals regulate their behavior by monitoring their progress and making adjustments as needed to stay on track.<br>3. Implementation: This component involves how individuals execute their actions based on their task representation and monitoring and control processes. It includes processes such as response selection, motor planning, and execution. The implementation component helps individuals carry out their intentions by selecting appropriate responses and executing them efficiently.<br><br>Together, these components work in an integrated manner to regulate behavior and enable individuals to adapt their behavior to changing situations. The M-PAC framework provides a comprehensive model for understanding how cognitive processes are involved in regulating behavior and has been used to explain a wide range of cognitive and behavioral phenomena in both laboratory and real-world settings.
Author	What is behaviour regulation?
ChatGPT	Behavior regulation refers to the process by which individuals control and modify their thoughts, feelings, and actions in response to internal and external stimuli. It involves the use of cognitive, emotional, and behavioral strategies to regulate one's behavior and achieve desired goals.<br><br>Behavior regulation can be both conscious and unconscious. Conscious regulation involves deliberate and intentional efforts to control one's behavior, such as setting goals, monitoring progress, and using self-control techniques. Unconscious regulation, on the other hand, involves automatic and spontaneous responses to stimuli, such as emotional reactions or habitual behaviors.<br><br>Behavior regulation is an important aspect of self-regulation, which is the ability to manage one's thoughts, feelings, and actions to achieve desired

	outcomes. It plays a critical role in many areas of life, including academic and professional success, interpersonal relationships, and physical and mental health.<br><br>Effective behavior regulation involves the ability to identify one's goals and priorities, monitor one's progress, adjust one's behavior when necessary, and use appropriate strategies to achieve desired outcomes. It requires a combination of self-awareness, self-control, and adaptability, and can be improved through practice and training.
Author	What is behaviour regulation for parents to support their child to be physically active?
ChatGPT	Behavior regulation for parents to support their child to be physically active involves using strategies and techniques to encourage and reinforce physical activity behaviors in children. Some examples of behavior regulation strategies that parents can use to support their child's physical activity include:<br><br>1. Setting goals: Parents can work with their child to set specific goals for physical activity, such as playing outside for a certain amount of time each day or participating in a specific sport or activity.<br>2. Providing incentives: Parents can provide rewards or incentives for their child's physical activity behavior, such as praise, stickers, or small prizes.<br>3. Modeling behavior: Parents can model physical activity behavior themselves, by engaging in physical activity alongside their child or participating in active family outings.<br>4. Creating a supportive environment: Parents can create an environment that supports physical activity, such as by providing opportunities for outdoor play or enrolling their child in sports or other physical activities.<br>5. Monitoring progress: Parents can monitor their child's physical activity behavior and provide feedback and support as needed to help their child achieve their goals.<br><br>By using these behavior regulation strategies, parents can help their child develop healthy physical activity habits that will support their overall health and well-being. It is important for parents to be consistent and persistent in their efforts to support their child's physical activity behavior, and to provide positive reinforcement and encouragement along the way.
Author	Write a fun and easy to understand article explaining the role of behaviour regulation for parents to support their child to be physically active
ChatGPT	As parents, we all want our children to be healthy and active, but getting them to move their bodies can sometimes feel like an uphill battle. That's where behavior regulation comes in! Behavior regulation refers to the

	process of controlling and modifying behavior in response to internal and external stimuli. In this case, we're talking about using behavior regulation strategies to encourage and reinforce physical activity behaviors in children.<br><br>So, what does behavior regulation look like when it comes to physical activity? Here are a few fun and easy strategies that parents can use to support their child's physical activity:<br><br>1. Set goals together: Sit down with your child and set specific physical activity goals, like playing outside for a certain amount of time each day or learning a new skill like jumping rope. This can help your child feel motivated and engaged in their physical activity.<br>2. Make it fun: Physical activity doesn't have to feel like a chore! Encourage your child to find activities they enjoy, whether it's playing tag with friends or practicing a new dance routine. When physical activity is fun, children are more likely to stick with it.<br>3. Provide incentives: Rewards and incentives can be a great way to encourage your child's physical activity. Try using a sticker chart or offering small prizes for reaching physical activity goals.<br>4. Be a role model: Children are more likely to be physically active if they see their parents being active too. Make physical activity a family affair by going on walks together or playing sports as a family.<br>5. Create a supportive environment: Make sure your child has access to safe and age-appropriate physical activity opportunities, whether that means signing them up for a sports team or providing outdoor play equipment in the backyard.<br><br>Remember, behavior regulation is all about helping your child develop healthy habits that will last a lifetime. By using these strategies to support your child's physical activity, you can help them stay healthy, active, and happy!
Author	Explain proactive and reactive tactics and provide bullet form examples
ChatGPT	Proactive tactics and reactive tactics are two different approaches to handling a situation or problem. Proactive tactics involve taking action before a problem occurs, while reactive tactics involve responding to a problem after it has already occurred.<br><br>Proactive tactics are often seen as more effective and efficient because they can prevent problems from occurring in the first place. Examples of proactive tactics include:<br><br>• Planning ahead: Anticipating potential problems and taking action to prevent them before they occur.

	• Preventative maintenance: Regularly maintaining equipment or systems to prevent breakdowns or failures.<br>• Risk management: Identifying potential risks and developing strategies to mitigate or avoid them.<br><br>On the other hand, reactive tactics involve responding to a problem after it has already occurred. While reactive tactics may be necessary in some situations, they are often seen as less effective and more costly than proactive tactics. Examples of reactive tactics include:<br><br>• Crisis management: Responding to a crisis or emergency situation after it has already occurred.<br>• Damage control: Trying to minimize the negative impact of a problem that has already happened.<br>• Remedial action: Taking action to correct a problem that has already occurred.<br><br>In general, proactive tactics are preferred because they can prevent problems from occurring in the first place, while reactive tactics are seen as less effective because they are often more costly and can't always fully resolve the issue.
Author	Write a fun and easy to understand summary of proactive strategies for parental support for child's physical activity
ChatGPT	If you're a parent who wants to support your child's physical activity, there are fun and easy ways to do it! Here are some tips:<br><br>1. Be a good role model: When you show your child that you enjoy physical activity, they'll be more likely to join in too.<br>2. Create opportunities: Make sure your child has access to fun physical activities, like playing outside or joining a sports team.<br>3. Set goals: Work with your child to set goals they can achieve, like learning to ride a bike or practicing a new skill.<br>4. Make it fun: Encourage your child to try different activities until they find something they enjoy.<br>5. Celebrate success: When your child reaches a goal, celebrate it! Maybe with a high five, a special treat or a fun activity.<br><br>By using these strategies, you can help your child develop healthy habits and enjoy being active!
Author	Write an easy to ready and interesting article about “How do I better plan ahead to help support my child to be physically active?”
ChatGPT	As a parent, you want your child to be healthy and active. However, with busy schedules and competing priorities, it can be challenging to prioritize physical activity. The key is to plan ahead and make physical activity a

	regular part of your family's routine. Here are some easy tips to help you better plan ahead and support your child to be physically active:<br><br>1. Schedule Physical Activity Time<br>To make physical activity a priority, schedule time for it just like you would for any other important activity. Consider scheduling a regular time each day for physical activity, such as after school or in the evening. This way, your child knows that physical activity is an important part of their routine.<br>2. Plan Active Family Activities<br>Planning fun and active family activities is a great way to spend time together while also promoting physical activity. Activities like hiking, biking, or playing a game of soccer in the park are fun and easy ways to get everyone moving.<br>3. Make Physical Activity Fun<br>One of the best ways to encourage physical activity is to make it fun. Involve your child in the planning process by asking them what activities they enjoy. Maybe they love playing basketball or dancing to music. Whatever it is, find ways to incorporate these activities into your family's routine.<br>4. Be a Role Model<br>Children often learn by example, so it's important for parents to model healthy habits. Make sure you're engaging in physical activity regularly and your child will be more likely to follow in your footsteps.<br>5. Be Flexible<br>Life can be unpredictable, and sometimes plans don't always go as expected. It's important to be flexible and adaptable when it comes to physical activity. If you can't stick to your regular routine, find other ways to incorporate physical activity into your day, such as taking a walk during lunch or doing a quick workout at home.<br><br>In conclusion, planning ahead is key to supporting your child's physical activity level. By scheduling physical activity time, planning active family activities, making physical activity fun, being a role model, and being flexible, you can help your child develop healthy habits that will benefit them for a lifetime. So, get planning and get moving!
Author	Provide a list of strategies to support my child to be physically active when I am stressed or busy.
ChatGPT	We all know that physical activity is important for our children's health and wellbeing. However, life can get busy and stressful, making it difficult to find the time and energy to support our children's physical activity levels. But fear not, there are still plenty of ways to help your child stay active, even when you're busy, stressed, or upset.<br><br>1. Make it a Game

	One fun way to support your child's physical activity when you're feeling stressed or busy is to turn it into a game. Challenge your child to a race or see who can do the most jumping jacks in 30 seconds. Not only will this get your child moving, but it can also help relieve stress and bring a smile to your faces.<br>2. Take the Scenic Route<br>When you're running errands or going to appointments, take the scenic route. This way, you can incorporate some physical activity into your day by taking a walk, riding bikes, or even playing a quick game of tag at the park. It's a win-win situation: you get to cross things off your to-do list while also supporting your child's physical activity level.<br>3. Prioritize Physical Activity<br>When your schedule is packed, it can be easy to let physical activity fall to the wayside. However, it's important to prioritize physical activity. Schedule it into your day just like you would any other appointment. Whether it's a quick 10-minute workout or a family walk after dinner, making physical activity a priority can help ensure it doesn't get forgotten.<br>4. Turn Chores into Exercise<br>Let's face it, chores are not usually fun. But what if you could turn them into a workout? Get your child involved in cleaning the house by turning it into a game. For example, see who can pick up the most toys in a minute or who can vacuum the fastest. Not only will this help your child stay active, but it can also help make chores more enjoyable for everyone.<br>5. Try a New Activity Together<br><br>When you're feeling stressed or upset, trying something new can be a great way to relieve stress and support your child's physical activity level. Sign up for a dance class, try a new sport, or take a yoga class together. Not only will you both get some physical activity, but you'll also have fun trying something new and bonding together.<br><br>In conclusion, supporting your child's physical activity level when you're busy, stressed, or upset can be a challenge. But by making it a game, taking the scenic route, prioritizing physical activity, turning chores into exercise, and trying new activities together, you can help ensure your child stays active and healthy even on your busiest and most stressful days.
Author	List the ways the environment influences reactive physical activity regulation
ChatGPT	The environment plays a crucial role in reactive physical activity regulation because it can influence the availability of opportunities for physical activity and the reinforcement of physical activity behaviors. Here are some ways in which the environment can influence reactive physical activity regulation:

	1. Access to Physical Activity Opportunities<br>The environment in which your child lives can impact their access to physical activity opportunities. For example, if there are no safe sidewalks or parks nearby, it may be difficult for your child to engage in physical activity. On the other hand, if there are plenty of parks, trails, and sports facilities nearby, it may be easier for your child to be physically active.<br>2. Social Norms<br>Social norms and cultural beliefs can also influence reactive physical activity regulation. If physical activity is not seen as important or valued in your child's social circle or culture, they may be less likely to engage in physical activity. Alternatively, if physical activity is highly valued and encouraged, your child may be more likely to engage in physical activity.<br>3. Reinforcement of Physical Activity Behaviors<br>The reinforcement of physical activity behaviors in the environment can also impact reactive physical activity regulation. If your child is consistently praised and rewarded for being physically active, they may be more likely to continue engaging in physical activity. However, if physical activity is not reinforced or is discouraged in the environment, your child may be less likely to engage in physical activity.<br>4. Access to Active Role Models<br>Having access to active role models in the environment can also influence reactive physical activity regulation. If your child sees friends, family members, or community members engaging in physical activity, they may be more likely to do so themselves. On the other hand, if there are no active role models in the environment, your child may be less motivated to engage in physical activity.<br><br>In conclusion, the environment can have a significant impact on reactive physical activity regulation. Access to physical activity opportunities, social norms, reinforcement of physical activity behaviors, and access to active role models are all factors that can influence your child's physical activity level. By understanding how the environment can influence physical activity, you can better support your child to be physically active and make positive changes in their environment to promote physical activity.
Author	List the ways social support can help parent support their child to be physically active.
ChatGPT	Social support can be a great asset for parents who want to support their child's physical activity. Here are some ways in which social support can help:<br><br>1. Encouragement and Motivation<br>Social support from family, friends, or coaches can provide encouragement and motivation for both parents and children to be

	physically active. Knowing that others support your efforts can help you stay on track and make physical activity a priority.<br>2. Accountability<br>Social support can also provide a sense of accountability. For example, if you plan to exercise with a friend or join a fitness class, you're more likely to show up and be consistent with your physical activity routine.<br>3. Role Modeling<br>Being around others who are physically active can also serve as positive role modeling for children. Seeing other children and adults being active can encourage children to follow their example and participate in physical activity themselves.<br>4. Sharing Ideas and Resources<br>Social support networks can also be a great source of information and resources. For example, other parents may have ideas for fun and creative ways to incorporate physical activity into daily routines or know of local community events and programs that promote physical activity.<br>5. Emotional Support<br>Finally, social support can provide emotional support during times of stress or difficulty. Knowing that you have a supportive network of people who understand and can provide advice and encouragement can help you and your child stay motivated and committed to physical activity.<br><br>In conclusion, social support can be a valuable tool for parents who want to support their child's physical activity. Encouragement and motivation, accountability, role modeling, sharing ideas and resources, and emotional support are all ways in which social support can help parents and children prioritize and maintain physical activity levels.
Author	What are the different types of social support?
ChatGPT	There are several types of social support that can be beneficial for individuals, including:<br><br>1. Emotional support: This type of support provides comfort, empathy, and understanding during stressful situations. It involves listening, offering advice, and providing encouragement to help the individual cope with their feelings.<br>2. Informational support: This type of support provides individuals with helpful information and resources. It involves providing advice, guidance, and knowledge to help the individual make informed decisions and solve problems.<br>3. Instrumental support: This type of support involves practical assistance and resources to help individuals achieve their goals. It can include help with transportation, child care, or other tasks that can make it easier for the individual to engage in physical activity.

	4. Appraisal support: This type of support provides individuals with feedback on their actions and behaviors. It involves offering constructive criticism, advice, and encouragement to help the individual improve their performance and achieve their goals.<br>5. Companionship support: This type of support involves providing individuals with a sense of belonging and connection. It involves spending time with others, engaging in social activities, and providing a sense of community and support.<br><br>Overall, social support can take many forms, and each type can be beneficial for individuals in different ways. By identifying the types of social support that are most helpful for them, individuals can create a supportive network that helps them achieve their goals, including staying physically active.
Author	Write a fun to read article to introduce parents the importance of social support in child physical activity level and strategies that they can use
ChatGPT	Are you struggling to motivate your child to stay physically active? Do you find it challenging to keep them engaged in sports and other activities? If so, you're not alone! Many parents face these challenges, but the good news is that social support can help!<br><br>What is social support, you ask? Social support is the assistance and encouragement that we receive from family, friends, and other individuals in our social network. In the context of physical activity, social support can play a crucial role in motivating and encouraging children to stay active and healthy.<br><br>Here are some strategies that you can use to tap into the power of social support and promote physical activity for your child:<br><br>1. Encourage participation in group activities: Joining a sports team, dance class, or other group activity can provide your child with the opportunity to interact with peers and receive positive reinforcement from their coaches and teammates.<br>2. Connect with other parents: Reach out to other parents in your community who are also interested in promoting physical activity for their children. You can share ideas and strategies, and even organize group activities or playdates that involve physical activity.<br>3. Use social media: Social media platforms can be a great way to connect with other parents and stay up-to-date on local events and activities related to physical activity.<br>4. Set goals together: Work with your child to set physical activity goals and track progress together. Celebrate achievements and use setbacks as opportunities for learning and growth.

	5. Celebrate success: Recognize and celebrate your child's achievements and progress in physical activity. Provide positive feedback and praise for their efforts and encourage them to continue to set new goals and strive for improvement.<br><br>In conclusion, social support can be a powerful tool in promoting physical activity for children. By tapping into the resources and support of your social network, you can create a supportive environment that encourages your child to stay active and healthy. Try out some of these strategies and see how social support can make a positive impact on your child's physical activity level!
Author	Provide a list of strategies to receive social support to help them encourage child to be physically active
ChatGPT	Here are some strategies for receiving social support to help encourage your child to be physically active:<br><br>1. Connect with other parents: Reach out to other parents in your community who share your interest in promoting physical activity for children. Attend local events and activities related to physical activity and strike up conversations with other parents. Joining parent groups, such as PTA or sport team parent groups, can be a great way to meet other like-minded parents.<br>2. Seek support from family and friends: Talk to family members and friends who can provide you with emotional support and encouragement. They can also help by providing child care, transportation, or even joining you and your child for physical activities.<br>3. Utilize online resources: The internet is full of resources that can help you connect with other parents and find ideas for promoting physical activity for children. Look for online communities and forums where parents can share ideas and support each other.<br>4. Engage with social media: Social media platforms can be a great way to connect with other parents and stay up-to-date on local events and activities related to physical activity. You can join parent groups, follow fitness influencers, or even create your own social media group for parents interested in promoting physical activity for children.<br>5. Work with professionals: Seek support from professionals, such as coaches, trainers, or pediatricians, who can provide you with guidance and advice on how to promote physical activity for your child.<br><br>Remember, social support can come in many forms, and it's important to find the type of support that works best for you and your child. By seeking support from family, friends, and professionals, you can create a

	supportive environment that encourages your child to be physically active and healthy.
Author	Based on the Multi-Process Action Control Framework, what types of parental support for physical activity are there?
ChatGPT	The multi-process action control framework suggests that there are three main types of parental support for child physical activity:<br><br>1. Instrumental support: This type of support involves providing tangible resources to support a child's physical activity, such as providing transportation to a sports practice or purchasing sports equipment.<br>2. Emotional support: This type of support involves providing encouragement, motivation, and positive feedback to a child to promote physical activity. Examples include expressing enthusiasm for a child's participation in sports or praising them for their efforts.<br>3. Autonomy-supportive support: This type of support involves promoting a child's sense of autonomy and competence in physical activity. This can involve allowing the child to make choices about the types of activities they participate in or providing opportunities for skill-building and mastery experiences.<br><br>Research has shown that all three types of parental support can be effective in promoting child physical activity, but autonomy-supportive support may be particularly important for fostering long-term engagement and enjoyment of physical activity.
Author	Provide examples of instrumental support for parents to improve child physical activity levels
ChatGPT	Sure, here are some examples of instrumental support that parents can use to improve their child's physical activity levels:<br><br>1. Provide transportation: Parents can offer to drive their child to sports practices, games, or other physical activity events if they are not within walking or biking distance.<br>2. Purchase equipment: Parents can buy sports equipment or gear to support their child's participation in physical activities, such as basketballs, soccer balls, or bicycles.<br>3. Enroll their child in classes or programs: Parents can enroll their child in sports leagues, dance classes, martial arts programs, or other physical activity programs to provide opportunities for regular physical activity.<br>4. Create a home gym or play area: Parents can create a home gym or play area with equipment such as jump ropes, hula hoops, or a mini trampoline to encourage their child to be physically active at home.

	5. Join in on physical activity: Parents can join in on physical activity with their child, such as taking family walks or bike rides, playing catch, or going to the park together.<br><br>These are just a few examples of instrumental support that parents can use to help their child be more physically active.
Author	Provide fun examples of regulatory support for parents to improve child physical activity.
ChatGPT	Sure, here are some fun examples of parental regulatory support for improving child physical activity:<br><br>1. Screen-time rules: Parents can set limits on the amount of time their child spends on electronic devices and encourage physical activity instead. For example, parents could set a rule that their child has to engage in 30 minutes of physical activity before they can watch TV or play video games.<br>2. Family fitness challenges: Parents can set up family fitness challenges, such as a weekly step-count challenge or a "plank-a-day" challenge. This can make physical activity fun and engaging for the whole family, while also encouraging healthy competition.<br>3. Creating an activity chart: Parents can create a chart that tracks their child's physical activity each day and provides rewards for meeting certain goals. For example, if a child engages in 60 minutes of physical activity each day for a week, they could earn a reward like a trip to the park or a healthy treat.<br>4. Active chores: Parents can assign physical activity-based chores around the house, such as raking leaves or shoveling snow, to encourage their child to be more active while also helping out with household tasks.<br>5. Fun physical activity games: Parents can encourage physical activity through fun games such as freeze tag, hopscotch, or a relay race in the backyard. These games can be enjoyed by the whole family and provide opportunities for active play.<br><br>Overall, there are many fun and creative ways that parents can use regulatory support to improve their child's physical activity levels.
Author	Provide fun examples of motivational support for parents to improve child physical activity
ChatGPT	Sure, here are some fun examples of parental motivational support for improving child physical activity:<br><br>1. Joining in the fun: Parents can encourage physical activity by joining in on the fun. For example, going for a family bike ride, playing catch,

	or shooting hoops together can be a fun and engaging way to promote physical activity.<br>2. Celebrating accomplishments: Parents can motivate their child to engage in physical activity by celebrating their accomplishments. For example, when a child reaches a certain number of steps or runs a certain distance, parents could celebrate with a special treat or activity.<br>3. Encouraging variety: Parents can encourage their child to engage in different types of physical activity to keep things fun and engaging. For example, parents could take their child to try out different sports or activities, such as swimming, dancing, or martial arts.<br>4. Setting achievable goals: Parents can help motivate their child by setting achievable goals that are specific and measurable. For example, setting a goal to run a certain distance or complete a certain number of steps each day can help motivate children to engage in physical activity.<br>5. Making it a family affair: Parents can motivate their child by making physical activity a family affair. For example, planning regular family hikes or walks, playing sports together, or participating in a charity walk or run can be a fun and engaging way to promote physical activity for the whole family.<br><br>Overall, there are many fun and creative ways that parents can provide motivational support to improve their child's physical activity levels, while also promoting a positive attitude towards exercise and making physical activity a fun and enjoyable part of family life.
Author	Provide some fun ways to help parents strengthen and improve their affective attitudes about supporting child physical activity.
ChatGPT	Sure, here are some fun ways to help parents strengthen and improve their affective attitudes about supporting child physical activity:<br><br>1. Try new activities: Experiment with different physical activities with your child until you find something that you both enjoy. It could be anything from hiking, to swimming, to jumping on a trampoline.<br>2. Make it a family affair: Get the whole family involved in physical activity by planning outings or vacations centered around physical activity. Take a family bike ride or hike in a nearby park, or plan a weekend camping trip.<br>3. Play games: Incorporate physical activity into your family game nights by playing games like Twister, charades, or tag.<br>4. Create a reward system: Set up a reward system for physical activity, such as a family outing or special treat, to help motivate and encourage everyone to stay active.

	5. Join a community: Join a local sports team or fitness group with other parents and their children. Not only will you get to be active with your child, but you'll also meet other like-minded parents and kids.<br>6. Get creative: Make physical activity fun by getting creative with it. Create obstacle courses in your backyard or play "Simon Says" with physical activity commands.<br>7. Use technology: There are many apps and games available that promote physical activity, such as dance games or fitness tracking apps. Use these tools to make physical activity more engaging and enjoyable for you and your child.<br><br>Remember, the most important thing is to find physical activities that you and your child enjoy and that fit into your lifestyle. By making physical activity fun and enjoyable, you'll strengthen your affective attitude towards it and create healthy habits that will benefit you and your child for years to come.
Author	Provide specific fun examples to improve affective attitude
ChatGPT	Sure, here are some specific fun examples to improve affective attitudes towards supporting child physical activity:<br>1. Dance Party: Have a dance party with your child, play their favorite music and dance around the living room with them. This is a great way to get your heart rate up and create positive feelings towards physical activity.<br>2. Backyard Games: Set up a variety of games in your backyard such as hopscotch, jump rope, or frisbee. This will not only provide a fun way to get active but also allow you to spend quality time with your child.<br>3. Adventure Walks: Take your child on an adventure walk, where you both can explore new places and take in the beauty of nature. This will not only provide exercise but also reduce stress and improve mood.<br>4. Beach Day: Go to the beach with your child and take part in activities such as swimming, beach volleyball, or building sandcastles. This will provide a fun way to exercise and soak up the sun.<br>5. Fun Run: Sign up for a fun run with your child, where you can both run, walk or jog for a good cause. This is a great way to create a positive attitude towards physical activity and instill a sense of community and giving back.<br>6. Indoor Trampoline Park: Take your child to an indoor trampoline park and bounce around together. This is a great way to get a full-body workout while having fun and laughing together.

	7. Home Workout Challenge: Create a home workout challenge with your child, where you can do exercises such as squats, push-ups, and lunges. This is a fun way to exercise and create positive feelings towards physical activity.<br><br>Remember, the key to improving affective attitudes towards physical activity is to make it enjoyable and fun. By trying out these specific fun examples, you'll be on your way to creating healthy habits that will last a lifetime.
Author	Write a fun to read article about the role of parental capabilities in support for child physical activity
ChatGPT	Do you remember the days when you used to run around the playground, play tag, and climb trees? It's no secret that physical activity is important for our health and wellbeing, but did you know that as a parent, you play a crucial role in supporting your child's physical activity? That's right - by improving your parental capabilities, you can help your child lead a healthy and active lifestyle.<br><br>Parental capabilities refer to the skills, resources, and attitudes that parents use to support their child's physical activity. There are many ways you can improve your parental capabilities and help your child stay active and healthy.<br><br>One of the most important ways to improve your parental capabilities is to provide instrumental support. This includes things like providing transportation to and from activities, buying sports equipment, and enrolling your child in a physical activity program. But let's be real - sometimes we need a little motivation to get moving too. Here are some fun ideas to help you provide instrumental support to your child:<br><br>• Turn physical activity into a family affair! Take a bike ride or go for a walk together.<br>• Make physical activity a regular part of your family's routine. Set aside a specific time for physical activity each day, whether it's a morning walk or an after-dinner game of catch.<br>• Encourage your child to try different activities until they find something they love. Maybe they'll enjoy dance classes or team sports, or maybe they'll prefer hiking or rock climbing. Keep trying new things until you find something that sticks.<br><br>Another important way to improve your parental capabilities is to provide regulatory support. This means creating an environment that supports physical activity. Here are some fun ideas to help you create a supportive environment:

	• Set limits on screen time and encourage your child to spend more time outdoors.<br>• Make healthy food choices together. Talk to your child about the importance of fueling their body with nutritious foods that will give them energy for physical activity.<br>• Create a space in your home where your child can be active, whether it's a designated play area or a backyard obstacle course.<br><br>Finally, motivational support is all about fostering a positive attitude towards physical activity. Here are some fun ideas to help you provide motivational support:<br><br>• Praise your child's efforts, not just their successes. Encourage them to keep trying, even when things get tough.<br>• Set goals together. Maybe your child wants to run a mile without stopping, or maybe they want to learn a new skill in their favorite sport. Celebrate their progress along the way.<br>• Create a fun challenge for your family, like a friendly competition or a scavenger hunt that involves physical activity.<br><br>Improving your parental capabilities can be fun and rewarding for both you and your child. By providing instrumental, regulatory, and motivational support, you can help your child lead a healthy and active lifestyle. So get moving and have fun!
Author	Provide specific fun examples to help parents improve their capabilities for supporting child physical activity
ChatGPT	Certainly! Here are some specific fun examples to help parents improve their capabilities for supporting child physical activity:<br><br>1. Instrumental Support:<br>• Have a weekly family game night that involves physical activity such as charades, Pictionary or dance party.<br>• Organize a scavenger hunt with physical activity challenges for your child and their friends.<br>• Plan a family hike or outdoor adventure at a nearby park or nature reserve.<br>2. Regulatory Support:<br>• Start a vegetable garden together and teach your child the importance of physical activity and nutrition for a healthy lifestyle.<br>• Use a fitness tracker or pedometer with your child to encourage them to walk more and set goals for daily steps.<br>• Plan a family bike ride or roller skating adventure on a weekend afternoon.

	3. Motivational Support:<br>• Make a fun reward system for reaching physical activity goals, such as a family outing to a local amusement park or water park.<br>• Watch sports games or videos of active events with your child and talk about the benefits of physical activity for overall health.<br>• Encourage your child to invite friends over for a game of soccer, basketball or other team sport to promote socialization and physical activity.<br><br>By incorporating these fun examples into your family's routine, you can improve your capabilities for supporting your child's physical activity and make healthy habits a fun and rewarding part of your family's lifestyle.
Author	Write a fun to read article about the role of opportunities for parents to support child physical activity
ChatGPT	Are you looking for ways to help your child get more active and live a healthy lifestyle? As a parent, one of the best things you can do is provide your child with plenty of opportunities to engage in physical activity. Here are some fun ways to do just that!<br><br>1. Sign Your Child Up for a Sports Team or Activity Club: Signing your child up for a sports team or activity club is a great way to provide them with structured physical activity opportunities. Whether it's soccer, basketball, gymnastics, or dance, there are plenty of options to choose from that will cater to your child's interests.<br>2. Make Physical Activity a Family Event: Get the whole family involved in physical activity by making it a fun and enjoyable event. Plan family hikes, bike rides, or even backyard games like tag, hide and seek, or hopscotch.<br>3. Incorporate Physical Activity into Your Daily Routine: Find ways to incorporate physical activity into your daily routine. For example, take the stairs instead of the elevator, walk or bike to school or work, or take a family walk after dinner.<br>4. Provide Access to Outdoor Spaces: Provide your child with access to outdoor spaces where they can run, play, and explore. This can be as simple as a backyard or a local park, but the more opportunities your child has to be outside, the more likely they are to engage in physical activity.<br>5. Create a Physical Activity Challenge: Challenge your child to a physical activity challenge. For example, you could challenge them to run a certain distance, ride their bike for a certain amount of time, or complete a certain number of push-ups. Make it fun and rewarding by offering incentives such as a family outing or special treat for completing the challenge.

	By providing your child with plenty of opportunities to engage in physical activity, you can help them develop healthy habits that will last a lifetime. So, whether it's signing them up for a sports team, planning family activities, or creating physical challenges, there are plenty of fun and effective ways to support your child's physical activity.
Author	Provide specific fun examples to help parents improve opportunities to support child physical activity
ChatGPT	Sure, here are some fun examples that parents can use to improve opportunities for their children to engage in physical activity:<br><br>1. Family Bike Rides: Plan a family bike ride once a week and explore new bike trails and parks. This can be a fun way for the whole family to get outside and enjoy the fresh air.<br>2. Dance Parties: Turn on some upbeat music and have a dance party with your kids. This can be done in the living room or backyard and is a great way to get moving and have fun together.<br>3. Scavenger Hunts: Create a scavenger hunt in the park or backyard with clues that involve physical activities such as jumping jacks or running sprints. This is a fun way to get kids moving while also keeping them entertained.<br>4. Obstacle Courses: Set up an obstacle course in the backyard or park using items such as cones, hula hoops, and jump ropes. Kids can work on their agility and balance while having fun.<br>5. Water Play: In the summer, set up a sprinkler or fill up a kiddie pool for kids to run through and splash around in. This is a great way to cool off and get some physical activity in.<br>6. Active Video Games: There are many video games available that require physical activity, such as dance games or sports games. This can be a fun way for kids to engage in physical activity while also playing their favorite video games.<br>7. Nature Walks: Take a nature walk with your children and encourage them to explore and collect items such as leaves and rocks. This is a great way to get outside and get some exercise while also learning about nature.<br><br>These are just a few examples of how parents can improve opportunities for their children to engage in physical activity. By making physical activity fun and enjoyable, parents can help their children develop healthy habits that will benefit them throughout their lives.
Author	Provide some more examples
ChatGPT	Sure, here are some additional fun examples to help parents improve opportunities for their children to engage in physical activity:

	1. Parkour: Encourage your child to practice parkour, a physical activity that involves jumping, climbing, and running through obstacles in an urban environment. This can be done in a park or backyard using objects such as picnic tables and benches.<br>2. Sports Leagues: Enroll your child in a local sports league such as soccer or basketball. This provides an opportunity for them to develop skills and engage in physical activity while also socializing with peers.<br>3. Gardening: Planting a garden and tending to it can be a fun way for kids to engage in physical activity while also learning about plants and nature.<br>4. Yoga: Practice yoga together as a family. This can help improve flexibility and balance while also promoting relaxation and stress relief.<br>5. Snow Sports: In the winter, take your child skiing or snowboarding. This is a great way to get outside and engage in physical activity while also enjoying the beauty of winter landscapes.<br>6. Trampoline Parks: Visit a trampoline park with your child for some bouncing fun. This provides an opportunity for kids to engage in physical activity while also improving balance and coordination.<br>7. Beach Activities: During the summer, take your child to the beach for some fun activities such as swimming, sandcastle building, and beach volleyball. This provides an opportunity to engage in physical activity while also enjoying the beach scenery.<br><br>These are just a few additional examples of how parents can improve opportunities for their children to engage in physical activity. By making physical activity fun and enjoyable, parents can help their children develop healthy habits that will benefit them throughout their lives.
Author	How do you make it fun for children when you're supporting them to be physically active?
ChatGPT	Making physical activity fun and enjoyable for children is key to encouraging them to be active and meet the recommended guidelines. Here are some tips to make physical activity fun for children:<br><br>1. Choose activities they enjoy: Let your child choose activities they enjoy and find fun. This could be anything from playing a sport, riding a bike, jumping rope, or dancing.<br>2. Make it a family affair: Plan physical activities that involve the whole family. This can make it more enjoyable for children and also create positive family memories.<br>3. Use games: Incorporate games into physical activities to make them more engaging and entertaining. For example, play tag, have relay races, or create an obstacle course.

	4. Set goals: Encourage your child to set physical activity goals for themselves and celebrate when they reach them. This can help them stay motivated and engaged.<br>5. Be creative: Try new activities and mix things up to keep it interesting. You can go for a nature walk, have a family dance party, or play a game of kickball.<br>6. Make it social: Encourage your child to invite friends to join in on physical activities. This can make it more enjoyable and create a positive social atmosphere.<br><br>Remember, physical activity doesn't have to be a chore or a boring task. By making it fun and enjoyable, you can help your child develop a lifelong love of physical activity and improve their overall health and wellbeing.
Author	Write an encouraging and fun message about the challenges of supporting child physical activity level.
ChatGPT	Hey there!<br>I just wanted to drop you a quick message to let you know that I think you're doing an amazing job supporting your child's physical activity level. I know it's not always easy, but your commitment and dedication are truly inspiring!<br>I also wanted to remind you that it's okay to face challenges along the way. Whether it's finding the time for physical activity or keeping your child motivated, know that you're not alone. We all face these challenges, and the important thing is that we keep pushing
Author	Write it in third person
ChatGPT	Hey there!<br>This is a message for all the parents out there who are working hard to support their child's physical activity level. It's important to acknowledge that it's not always easy, but their commitment and dedication are truly inspiring!<br>It's okay to face challenges along the way. Whether it's finding the time for physical activity or keeping their child motivated, know that they're not alone. We all face these challenges, and the important thing is that they keep pushing forward.<br>Remember, every little bit counts. Even if it's just a quick game of catch or a family walk around the neighborhood, every opportunity for physical activity is a step in the right direction.<br>So keep up the great work, parents! Your efforts are making a positive impact on your child's health and wellbeing.
Author	Based on the multi-process action control framework, explain the motivational support that a parent can provide for a child to improve physical activity. Please provide examples activities and goal-setting activities.

ChatGPT	The Multi-Process Action Control (M-PAC) framework proposes that behavior is regulated by both motivational and volitional processes. Motivational processes involve the desire to engage in a behavior, while volitional processes involve the ability to execute the behavior. Therefore, in order to increase a child's physical activity, a parent can provide both motivational and volitional support.<br>Motivational support involves encouraging a child's desire to engage in physical activity. This can be achieved by setting goals, providing positive feedback, and using rewards. Here are some examples of motivational support a parent can provide:<br>1. Goal-Setting Activities: Encourage your child to set specific physical activity goals, such as walking for 30 minutes a day, or practicing a specific sport for a certain amount of time. Help them create a plan to achieve these goals and track their progress.<br>2. Positive Feedback: Provide positive feedback for your child's physical activity efforts, such as telling them how proud you are of their progress or commenting on how their physical activity is making them stronger and healthier.<br>3. Rewards: Offer rewards for achieving physical activity goals, such as a special outing or activity or even a small prize. Make sure the rewards align with the goals and help reinforce the behavior.<br>4. Role Model: Be a good role model by engaging in physical activity yourself, and involving your child in these activities. This will help your child see the importance of physical activity and encourage them to participate.<br>5. Social Support: Encourage your child to participate in physical activity with their peers or in organized activities, such as a sports team. This will provide social support and help make physical activity more enjoyable.<br>Some examples of physical activities that a child can engage in include running, swimming, cycling, playing a sport, dancing, or even simply taking a walk. By providing motivational support and encouraging physical activity, a parent can help their child develop healthy habits and a positive attitude towards physical activity.
Author	Write a blog to help explain and encourage parents about the importance of parental support for child physical activity.
ChatGPT	Title: The Critical Role of Parental Support in Promoting Child Physical Activity<br><br>Physical activity is an essential component of a child's healthy development. Engaging in regular physical activity can help children maintain a healthy weight, improve their cardiovascular health, build strong bones and muscles, and reduce their risk of developing chronic diseases. Despite these benefits, many children today do not get enough

	physical activity. In this blog, we will explore the critical role of parental support in promoting child physical activity.<br><br>As a parent, you play a significant role in supporting your child's physical activity. Here are some reasons why parental support is crucial:<br><br>1. Children need motivation: Children need encouragement and motivation to engage in physical activity. As a parent, you can provide your child with motivational support by expressing enthusiasm for their physical activity, praising their efforts, and setting realistic goals.<br>2. Children need resources and opportunities: Children also need the resources and opportunities necessary to engage in physical activity. As a parent, you can provide your child with instrumental support by providing transportation to sports practices or games, purchasing sports equipment, or enrolling them in organized sports programs.<br>3. Children need guidance and structure: Children also need guidance and structure to engage in physical activity consistently. As a parent, you can provide your child with regulatory support by setting rules and expectations for their physical activity behaviors. This may include setting limits on screen time or requiring your child to engage in physical activity for a certain amount of time each day.<br><br>In addition to these reasons, research has shown that parental support is a critical factor in promoting children's physical activity behaviors. Studies have found that children who receive support from their parents are more likely to engage in regular physical activity and maintain healthy lifestyles as they grow older.<br><br>So, how can you support your child's physical activity? Here are some ideas:<br><br>1. Make physical activity a family affair: Instead of sitting on the couch watching TV, get outside and be active as a family. Take a walk, go for a bike ride, or play a game of tag.<br>2. Encourage your child to try different activities: Your child may not enjoy traditional sports, but that doesn't mean they can't be physically active. Encourage your child to try different activities, such as dance, yoga, or martial arts.<br>3. Set aside dedicated time for physical activity: Schedule time for physical activity into your family's daily routine. This can be as simple as taking a walk after dinner or going for a bike ride on the weekends.

	In conclusion, parental support is a critical factor in promoting child physical activity. By providing your child with motivation, resources, and guidance, you can help them develop healthy physical activity habits that will benefit them for a lifetime. So, take an active role in supporting your child's physical activity behaviors and help them lead a healthy and active life.
Author	How can families create habits to support your child's physical activity? What are some of the cues? List them.
ChatGPT	Creating habits to support a child's physical activity can help make it a regular part of their daily routine. Here are some strategies families can use to create habits that support their child's physical activity:<br><br>1. Schedule it: Scheduling physical activity into a regular routine can help make it a habit. For example, setting aside time each day for physical activity, such as after school or before dinner.<br>2. Set reminders: Using reminders, such as phone alarms or a family calendar, can help cue the family to engage in physical activity.<br>3. Make it enjoyable: By making physical activity enjoyable, children are more likely to want to engage in it regularly. Incorporating games or other fun activities into physical activity can help make it more enjoyable.<br>4. Use visual cues: Placing visual cues around the house, such as a chart or a sign, can serve as a reminder to engage in physical activity.<br>5. Involve the family: By involving the entire family in physical activity, it becomes a shared experience and can help make it a habit for everyone.<br>6. Be consistent: Consistently engaging in physical activity is key to creating a habit. By sticking to a regular routine, it becomes easier to maintain over time.<br>7. Celebrate progress: Celebrating progress and achievements, such as reaching a new fitness goal or completing a physical challenge, can help motivate children to continue engaging in physical activity.<br><br>By incorporating these strategies and using cues to remind and encourage physical activity, families can establish habits that support their child's physical activity and promote a healthy and active lifestyle.
Author	What are some of the strategies to become physically active as a family?
ChatGPT	Staying physically active as a family can be a fun and engaging way to promote a healthy and active lifestyle. Here are some strategies to stay physically active as a family:

	1. Set regular physical activity goals: Set regular physical activity goals as a family, such as taking a daily walk after dinner or participating in a weekend activity together.<br>2. Choose activities that everyone enjoys: Choose physical activities that are enjoyable for everyone in the family, such as hiking, biking, or swimming.<br>3. Make physical activity a part of your routine: Schedule physical activity as a regular part of your family's routine, such as setting aside time each week for a family activity.<br>4. Be active together: Participate in physical activity together as a family, such as playing sports or taking a fitness class.<br>5. Incorporate physical activity into daily life: Look for opportunities to incorporate physical activity into daily life, such as walking or biking instead of driving or taking the stairs instead of the elevator.<br>6. Celebrate accomplishments: Celebrate accomplishments and milestones as a family, such as reaching a fitness goal or completing a challenging physical activity.<br>7. Be supportive: Support each other in achieving physical activity goals and encourage one another to stay active.<br><br>By using these strategies, families can stay physically active together and promote a healthy and active lifestyle for everyone.
